# The assessment of capacity limitations in psychiatric work disability evaluations by the social functioning scale Mini-ICF-APP

**DOI:** 10.1186/s12888-021-03467-w

**Published:** 2021-09-30

**Authors:** Timm Rosburg, Regina Kunz, Bruno Trezzini, Urban Schwegler, Jörg Jeger

**Affiliations:** 1 Department of Clinical Research, EbIM Research & Education, University of Basel Hospital, Basel, Switzerland; 2 Swiss Paraplegic Research, Nottwil, Switzerland; 3 Department of Health Sciences and Medicine, University of Lucerne, Lucerne, Switzerland; 4 MEDAS Zentralschweiz, Lucerne, Switzerland

**Keywords:** Insurance medicine, Social security system, Disability insurance, Medical assessment, Psychiatry, Disability benefits, Work capacity

## Abstract

**Objective:**

Insurers frequently commission medical experts to estimate the degree of the remaining work capacity (RWC) in claimants for disability benefits. The social functioning scale *Mini-ICF-APP* allows for a rating of activity and participation limitations in thirteen capacity domains, considered as particularly relevant for work ability. The current study sought to evaluate the role of the Mini-ICF-APP ratings in psychiatric work disability evaluations, by examining how the capacity limitation ratings varied with the claimants’ primary psychiatric diagnoses and how the ratings were related to RWC estimates.

**Methods:**

Medical experts estimated the RWC of 946 claimants with mental disorders and rated their activity and participation limitations using the Mini-ICF-APP, with higher ratings reflecting more severe limitations. The ratings were compared between claimants with different psychiatric diagnoses by analyses of variance. The mean Mini-ICF-APP rating across all capacity domains as well as all capacity-specific ratings were entered in simple or multiple regression models to predict the RWC in an alternative job.

**Results:**

The Mini-ICF-APP capacity limitation ratings in all domains but mobility were higher for claimants with personality and behavior disorders as compared to those with mood disorders or with neurotic, stress-related and somatoform disorders. The largest differences were observed in social capacities (e.g. group integration: F _2, 847_ = 78.300, *P* < 0.001). In claimants with depression, all ratings increased with the severity of the diagnosis (all Fs _2, 203_ > 16.393, all Ps < 0.001). In the overall sample, the mean Mini-ICF-APP rating showed a strong negative correlation with the estimated RWC (r = −.720, *P* < 0.001). Adding the capacity-specific ratings to the prediction model improved this prediction only marginally.

**Discussion:**

The Mini-ICF-APP allows for documenting claimants’ activity and participation limitations, which is likely to increase the transparency of medical experts’ RWC estimates and enables them to check the plausibility of such estimates. However, our study showed that despite the strong association between RWC and Mini-ICF-APP ratings, half of the RWC variance was unrelated to the capacity limitations documented in the Mini-ICF-APP.

**Supplementary Information:**

The online version contains supplementary material available at 10.1186/s12888-021-03467-w.

## Introduction

Statutory disability insurances provide disability benefits for individuals with strong restrictions in their work capacity due to disease- or injury-related impairments, in case the work capacity cannot be restored or improved by vocational integration services. In 2019, the Swiss disability insurance paid disability benefits of 5.4 billion CHF (5.7 billion US $) to a total of 247′000 claimants [1]. The latter number corresponds to 4% of all insured Swiss citizens. In Switzerland, the rate of individuals receiving disability benefits is thus higher than the rate of the unemployed [2].

Work disability assessments by medical experts are internationally the most common procedure to determine claimants’ entitlement for benefits [3]. The definition of the expert’s role in these assessments depends on the national social security system. In Switzerland, medical experts assess the claimants’ remaining work capacity (RWC) in percent, as a basis for determining claimants’ eligibility for disability benefits and the amount of benefits they are entitled to. There are currently four levels of disability benefits, varying by percentage of *invalidity (“Invalidität*”). In order to qualify for such benefits, claimants must suffer from at least 40% invalidity, defined as a 40% income loss due to permanent work incapacity in relation to the income they could achieve without a disability. An invalidity of 40% qualifies for 25% of full disability benefits (“*Viertelsrente*”). Beyond 40% invalidity, there is a 25% increase of full disability benefits with a 10% increase of invalidity. This means that claimants with 50, 60, and 70% invalidity receive 50, 75, and 100% of full disability benefits, respectively [4].

These numbers suggest that small variance in the estimated RWC can have considerable impact on the granting of disability benefits. Ideally, the estimation of RWC should primarily depend on claimants’ health-related impairments. Unfortunately, the interrater agreement in RWC estimates is commonly limited even for the very same claimants [5–7], which might ultimately result in unjust financial compensation. Pizala [8] criticized that, in particular, psychiatric work disability evaluations often lack information on what considerations RWC estimates are based on, implying an insufficient transparency and a lack of objective data in these evaluations.

One way to tackle such a shortcoming is the implementation of standardized instruments in work disability evaluations that a) document activity and participation limitations of claimants, and b) form an empirical basis for the RWC estimated by the medical expert. Instruments for assessing work disability in a quantitative way are still sparse, albeit the production of core sets derived from the International Classification of Functioning, Disability and Health, ICF [9] have created a basis for changing the landscape of instruments in work disability assessments, but also provided a taxonomic framework for job placement and vocational rehabilitation [10–13].

The social functioning scale Mini-ICF-APP represents a rating instrument for activity and participation limitations of individuals with mental disorders [14–16] and is increasingly used in work disability evaluations. The name *Mini-ICF-APP* reflects the fact that the to-be-rated activity and participation domains were derived from the ICF [9], whereby APP stands for activity and participation limitations in mental/psychiatric disorders. The Mini-ICF-APP encompasses ratings of thirteen different capacity domains, which are considered as particularly relevant for work participation, namely (I) adherence to regulations, (II) planning and structuring of tasks, (III) flexibility, (IV) applying expertise, (V) competence to judge and decide, (VI) endurance, (VII) assertiveness, (VIII) contact with others, (IX) group integration, (X) intimate relationships, (XI) non-work activities, (XII) self-care, and (XIII) mobility. The ratings allow the differentiation of five levels of limitations, numerically coded from “0” to “4”. These levels are a) no disability (0 to 4% incapacity), b) mild disability (5 to 24% incapacity), c) moderate disability (25 to 49% incapacity), d) severe disability (50 to 95% incapacity), e) total disability (96 to 100% incapacity, [14]). The Mini-ICF-APP is available in several languages, including German, English, French, Italian, and Polish [14–19].

The role of the Mini-ICF-APP in work disability evaluation is so far little investigated. The current study focused on two important aspects, namely the question whether the Mini-ICF-APP ratings can capture diagnosis-specific capacity limitations and the question how the ratings relate to RWC estimates. With regard to the first aspect, previous research suggested that the nature of the mental disorder has some impact on the extent of activity and participation limitations. An Italian community-based study revealed larger Mini-ICF-APP sum scores in patients with schizophrenia than in patients with major depression and larger sum scores in patients with major depression than in patients with anxiety disorders [19]. Such findings presumably reflect in parts the differential severity of mental disorders, with increasing severity of mental disorders associated with more capacity limitations and higher Mini-ICF-APP total scores [19, 20]. However, such findings may partly also reflect disorder-specific capacity limitations, which has been investigated only to minimal extent so far (for different limitations in different work anxieties, see [21]). As one study aim, we sought to reveal such disorder-specific capacity limitations. To this end, we contrasted the ratings in each capacity domain and across all capacity domains between claimants for disability benefits with a) mood disorders, b) neurotic, stress-related and somatoform disorders, and c) disorders of adult personality and behavior. Moreover, for mood disorders, we compared the Mini-ICF-APP ratings of patients with mild, moderate and severe depressive episodes in order to test whether activity and participation limitations would increase with the severity of the disorder.

The second and major aim of the current study was to investigate the association between RWC estimates and Mini-ICF-APP ratings, which was previously addressed in two studies, including an own one. In both previous studies, the same psychiatrist rated their activity and participation limitations and estimated the claimants’ RWC. In a sample of 447 claimants for disability benefits, we showed that the RWC estimates and Mini-ICF-APP ratings agreed, as to-be-expected, on a group level: claimants with high RWC showed low levels of capacity limitations and claimants with low RWC showed high levels of limitations [22]. Assessing this kind of agreement in a sample of 121 claimants in more detail, Habermeyer and colleagues [23] revealed a significant correlation of r = 0.663 between work disability (as complement to RWC) and the Mini-ICF-APP capacity limitation sum score. However, up to now, it is unknown whether primarily the global functional disability (as reflected in the Mini-ICF-APP sum score or its respective mean score) contributes to the RWC estimate, or whether some domains of activity and participation are of higher relevance for medical experts when estimating the RWC. To answer this question, we calculated several linear regression models with the Mini-ICF-APP mean score, ratings in individual domains, or both as predictors for the RWC and compared how well these models explained the RWC variance. Moreover, in extension to our previous study [22], we contrasted the Mini-ICF-APP ratings between claimants with high, moderate, and low RWC.

## Methods

### Participants and data collection

From February 2010 to October 2016, 946 claimants for disability benefits undergoing a multidisciplinary work disability evaluation at MEDAS Zentralschweiz (Lucerne, Switzerland) were rated with the Mini-ICF-APP as part of their psychiatric assessment. This included 447 claimants of our previous study [22]. The mean age of the claimants (532 female, 414 male) was 48.9 years (SD 8.5 years). Details on the distribution of gender and age across in the major psychiatric diagnoses can be found in Supplementary Table S1. Only claimants who underwent a psychiatric evaluation and received an ICD-10 diagnosis from the chapter “F – Mental and behavioural disorders” were included. The same psychiatrists conducted the Mini-ICF-APP ratings as part of their evaluation and estimated the RWC resulting from mental disorders. The pool of raters consisted of seven psychiatrists, three of whom conducted 84% of all ratings. For six individuals, the experts refrained from estimating the RWC. Consequently, the regression analyses for predicting the RWC were based on 940 cases only.

### Statistical analyses

Due to data protection regulations, we only analysed pooled data and did not differentiate between the psychiatrists (due to their limited number, the anonymity of the medical experts would not have been guaranteed). In addition to the ratings in each of the 13 domains, we calculated the average rating across domains (*MICF*_*mean*_) as a measure for global capacity limitation. Linden et al. [15] named this average rating ‘*global value*’. Descriptive statistics on the summed MICF rating (*MICF*_*total*_) were also calculated. In analogy to the International Classification of Functioning [9], the ratings in each domain range from “0” to “4”, with higher ratings corresponding to more severe activity and participation limitations. Mini-ICF-APP data are often not normally distributed, but tend to show a right skew. The analysis of variance (ANOVA), used for analysing group differences, provides relatively robust results for data that are not normally distributed [24].

First, we compared all Mini-ICF-APP ratings in a univariate ANOVA between the three major groups of mental disorders as psychiatric diagnosis. Based on the ICD-10 (https://icd.who.int/browse10/2019/en#/V), these groups were (a) mood disorders (F30-F39), (b) neurotic, stress-related and somatoform disorders (‘*neurotic disorders*’, F40-F48), and (c) disorders of adult personality and behavior (*‘personality disorders’*, F60-F69). Moreover, for mood disorders, we compared the ratings in another univariate ANOVA between patients with mild (F32.0), moderate (F32.1), and severe (F32.2) depressive episodes. Inclusion of age and sex as co-variables had no relevant impact on the ANOVA results. For the sake of brevity, age and sex were therefore not considered as co-variables. The *p* values as reported in Tables 1 to 3 were not corrected for multiple testing. All significant F values were followed by post-hoc least significant difference tests, with an α criterion of *P* = 0.01. The F statistics provided identical results for MICF_mean_ and MICF_total_, as one value can be linearly derived from the other (MICF_total_ = k * MICF_mean_; with k as the number of domains = 13).

**Table 1:** Mini-ICF-APP ratings of claimants with different psychiatric diagnoses

	a) F30-F39 Mood disorders	b) F40-F48 Neurotic disorders	c) F60-F69 Personality disorders	Effects of diagnosis
Total N	359	347	142	
Female/male	192/167	218/129	80/62	Χ^2^ = 6.459, *P* = 0.040
Mean age	50.1 (7.7)	48.9 (8.5)	47.4 (8.8)	F = 6.037, *P* = 0.002 a > c
(1) adherence to regulations	1.39 (0.92)	1.15 (0.91)	1.84 (0.96)	F = 28.588, *P* < 0.001 c > a > b
(2) planning and structuring of tasks	1.56 (0.88)	1.21 (0.90)	1.52 (0.94)	F = 14.191, *P* < 0.001 c, a > b
(3) flexibility	2.04 (0.72)	1.72 (0.88)	2.29 (0.79)	F = 29.725, P < 0.001 c > a > b
(4) applying expertise	1.33 (0.85)	1.14 (0.92)	1.44 (1.01)	F = 6.995, *P* = 0.001 c, a > b
(5) competence to judge and decide	1.59 (0.96)	1.19 (0.93)	1.35 (1.08)	F = 14.708, P < 0.001 a > b
(6) endurance	2.19 (0.67)	2.11 (0.73)	2.34 (0.84)	F = 5.255, *P* = 0.005 c > b
(7) assertiveness	1.85 (0.82)	1.37 (0.93)	1.85 (0.90)	F = 30.595, P < 0.001 c, a > b
(8) contact with others	1.39 (0.86)	1.03 (0.84)	1.98 (0.88)	F = 63.462, P < 0.001 c > a > b
(9) group integration	1.39 (0.89)	1.18 (0.89)	2.26 (0.80)	F = 78.300, P < 0.001 c > a > b
(10) intimate relationships	1.29 (0.84)	1.07 (0.91)	1.78 (0.91)	F = 32.380, P < 0.001 c > a > b
(11) non-work activities	1.57 (0.84)	1.45 (0.82)	1.83 (0.90)	F = 10.420, P < 0.001 c > a, b
(12) self-care	0.29 (0.59)	0.23 (0.53)	0.42 (0.68)	F = 5.294, P = 0.005 c > b
(13) mobility	0.81 (0.96)	0.75 (0.87)	0.71 (0.94)	F = 0.730, n.s.
MICF_mean_	1.44 (0.59)	1.20 (0.57)	1.66 (0.55)	F = 36.087, P < 0.001 c > a > b
MICF_total_	18.7 (7.6)	15.6 (7.5)	21.6 (7.2)	
RWC ≤ 30%	20.2%	16.2%	45.7%	Χ^2^ = 56.218, P < 0.001
RWC > 30, < 70%	45.7%	44.8%	37.1%	
RWC ≥70%	34.2%	39.0%	17.1%	

The right column (‘Effects of Diagnosis’) shows the results of the statistical comparison between the three sub-samples, with significant post-hoc tests indexed by greater-than signs. The three top rows display the number of claimants in each sub-sample (N), the number of female and male claimants, and the mean age. The following rows show the Mini-ICF-APP ratings in each capacity domain, as well as the two global capacity ratings (MICFmean and MICFtotal). The numbers in parentheses refer to the standard deviations (SDs). The bottom row lists the percentage of claimants with low, moderate, and high RWC in an alternative job. RWC estimates of six claimants were missing. Claimants with personality disorders had the highest MICFmean as well as the highest percentage of claimants with low RWC

**Table 2:** Mini-ICF-APP ratings of claimants with depressive episodes

	a) F32.0 Mild depressive episodes	b) F32.1 Moderate depressive episodes	c) F32.2 Severe depressive episodes	Effects of severity
Total N	58	112	34	
Female/male	25/33	60/52	21/13	Χ^2^ = 3.249, n.s.
Mean age	51.3 (7.4)	49.8 (7.3)	47.7 (7.3)	F = 2.637, n.s.
(1) adherence to regulations	0.66 (0.69)	1.46 (0.76)	1.94 (1.07)	F = 31.707, *P* < 0.001 c > b > a
(2) planning and structuring of tasks	0.91 (0.82)	1.60 (0.63)	2.21 (0.85)	F = 35.627, *P* < 0.001 c > b > a
(3) flexibility	1.62 (0.67)	2.12 (0.47)	2.65 (0.70)	F = 34.937, P < 0.001 c > b > a
(4) applying expertise	0.85 (0.64)	1.42 (0.71)	2.02 (0.94)	F = 28.066, P < 0.001 c > b > a
(5) competence to judge and decide	1.04 (0.91)	1.67 (0.77)	2.38 (0.94)	F = 25.847, P < 0.001 c > b > a
(6) endurance	1.82 (0.55)	2.18 (0.54)	2.74 (0.61)	F = 29.244, P < 0.001 c > b > a
(7) assertiveness	1.50 (0.78)	1.89 (0.58)	2.29 (0.69)	F = 15.973, P < 0.001 c > b > a
(8) contact with others	0.85 (0.74)	1.46 (0.66)	2.12 (0.77)	F = 36.068, P < 0.001 c > b > a
(9) group integration	0.85 (0.74)	1.50 (0.73)	2.04 (0.86)	F = 28.729, P < 0.001 c > b > a
(10) intimate relationships	0.97 (0.75)	1.48 (0.73)	1.84 (0.80)	F = 16.393, P < 0.001 c, b > a
(11) non-work activities	0.91 (0.73)	1.65 (0.65)	2.27 (0.77)	F = 43.464, P < 0.001 c > b > a
(12) self-care	0.05 (0.22)	0.25 (0.51)	0.75 (0.82)	F = 19.798, P < 0.001 c > b, a
(13) mobility	0.16 (0.41)	0.84 (0.99)	1.41 (1.05)	F = 23.613, P < 0.001 c > b > a
MICF_mean_	0.94 (0.41)	1.50 (0.35)	2.04 (0.55)	F = 81.953, P < 0.001 c > b > a
MICF_total_	12.2 (5.3)	19.5 (4.6)	26.5 (7.2)	
RWC ≤ 30%	0.0%	11.8%	67.6%	Χ^2^ = 148.109, P < 0.001
RWC > 30, < 70%	19.0%	73.6%	26.5%	
RWC ≥70%	81.0%	14.5%	5.9%	

The right column (‘Effects of Severity’) shows the results of the statistical comparison between the three sub-samples, with significant post-hoc tests indexed by greater-than signs. The three top rows display the number of claimants in each sub-sample (N), the number of female and male claimants, and the mean age. The following rows show the Mini-ICF-APP ratings in each capacity domain, as well as the two global capacity ratings (MICFmean and MICFtotal). The numbers in parentheses refer to the standard deviations (SDs). RWC estimates of two claimants were missing. Claimants with severe depressive episodes had the highest MICFmean as well as the highest percentage of claimants with low RWC

**Table 3:** Mini-ICF-APP ratings in claimants with high, moderate, and low RWC

Subsamples defined by RWC	a) High RWC RWC ≥ 70%	b) Moderate RWC 30% < RWC < 70%	c) Low RWC RWC ≤ 30%	Effects of RWC categorization
N	307	402	231	
Female/male	161/146	230/172	135/96	Χ^2^ = 2.375, n.s.
Mean age	49.7 (8.1)	49.2 (8.4)	47.6 (8.9)	F = 4.399, *P* = 0.013
(1) adherence to regulations	0.81 (0.78)	1.43 (0.79)	2.10 (0.88)	F = 169.333, *P* < 0.001 a > b > c
(2) planning and structuring of tasks	0.90 (0.81)	1.48 (0.73)	2.14 (0.82)	F = 167.835, P < 0.001 a > b > c
(3) flexibility	1.45 (0.82)	1.99 (0.63)	2.57 (0.68)	F = 165.443, P < 0.001 a > b > c
(4) applying expertise	0.80 (0.77)	1.32 (0.79)	1.96 (0.82)	F = 136.008, P < 0.001 a > b > c
(5) competence to judge and decide	1.03 (0.88)	1.46 (0.90)	1.90 (1.05)	F = 56.346, P < 0.001 a > b > c
(6) endurance	1.71 (0.71)	2.24 (0.59)	2.67 (0.65)	F = 148.684, P < 0.001 a > b > c
(7) assertiveness	1.20 (0.83)	1.72 (0.81)	2.24 (0.78)	F = 109.625, P < 0.001 a > b > c
(8) contact with others	0.82 (0.77)	1.33 (0.79)	2.07 (0.85)	F = 163.016, P < 0.001 a > b > c
(9) group integration	0.87 (0.76)	1.49 (0.84)	2.21 (0.83)	F = 182.238, P < 0.001 a > b > c
(10) intimate relationships	0.78 (0.77)	1.27 (0.80)	1.96 (0.81)	F = 145.214, P < 0.001 a > b > c
(11) non-work activities	1.05 (0.76)	1.63 (0.71)	2.12 (0.79)	F = 136.452, P < 0.001 a > b > c
(12) self-care	0.11 (0.35)	0.22 (0.48)	0.70 (0.85)	F = 78.266, P < 0.001 a, b > c
(13) mobility	0.44 (0.70)	0.83 (0.89)	1.12 (1.07)	F = 40.168, P < 0.001 a > b > c
MICF_mean_	0.92 (0.48)	1.42 (0.40)	1.98 (0.48)	F = 370.865, P < 0.001 a > b > c
MICF_total_	12.0 (6.3)	18.4 (5.1)	25.8 (6.3)	

The right column (‘Effects of RWC categorization’) shows the results of the statistical comparison between the three sub-samples, with significant post-hoc tests indexed by greater-than signs. All Mini-ICF-APP ratings increased with decreasing RWC

The psychiatric experts estimated the RWC in the last and in an alternative job on the general labor market. An alternative job considers the claimant’s disabilities and accounts for adjustments to compensate them. This adjustment lowers the impact of disabilities on the work capacity and is used by the insurer to determine invalidity. We sought to reveal how the expert’s estimate of the RWC in an alternative job related to the Mini-ICF-APP ratings. For this purpose, we first compared the Mini-ICF-APP ratings in a univariate ANOVA between claimants with low, moderate, and high RWC (poor: 30% or less RWC; moderate: RWC between > 30 and < 70%; high: RWC of 70% or above). This approach extends the one by Jeger et al. [22] who exclusively contrasted the low and high RWC groups. Second, we used the Mini-ICF-APP ratings for predicting the RWC estimates by the medical expert. To this end, we ran four different kinds of linear regressions. A) In a simple linear regression analysis, we used the average Mini-ICF-APP rating (MICF_mean_) as predictor. B) In a multiple stepwise regression analysis, we used the domain-specific Mini-ICF-APP ratings as predictors, without considering MICF_mean_. C) In a hierarchical multiple regression analysis, we used MICF_mean_ in the first step and, in a second step, we determined which domain-specific Mini-ICF-APP ratings would further improve the prediction of the RWC. D) In a final univariate regression analysis, we swapped the dependent and independent variable of the first simple linear regression analysis and used the RWC for predicting the average Mini-ICF-APP ratings. The latter analysis was conducted in order to overcome limitations of the other regression models due to the (‘bounded’) data distribution of RWC, as detailed in the results section.

## Results

### Mini-ICF-APP ratings: effects of the psychiatric diagnosis

848 of the 946 participants had either a mood disorder (F30-F39), a neurotic disorder (F40-F48), or a personality disorder (F60-F69) as primary psychiatric diagnosis. The distribution of the participants across these diagnoses, including information about their age and sex, is provided in Table 1. Due to their insufficient sample sizes, participants with other primary diagnoses were not considered in this analysis. Descriptive Mini-ICF-APP data on the total sample can be found in Supplementary Tables S2 and S3.

The univariate ANOVA with diagnosis as between-subject factor revealed significant differences in all domains but mobility between the three groups of psychiatric disorders (F30-F39 vs. F40-F48 vs. F60-F69). Claimants with personality disorders were on average more limited in activity and participation as compared to claimants with one of the other two disorders; claimants with mood disorders were on average more limited than claimants with neurotic disorders (Table 1**,** MICF_mean_). The percentage of RWC estimates ≤30% was also highest among claimants with personality disorders (Table 1, bottom row). Across all three sub-samples, limitations in the capacity domains flexibility and endurance were most pronounced, as compared to other capacity domains. Claimants with personality disorder showed additionally pronounced limitations in their social functioning (contact with others, group integration, intimate relationships, adherence to regulations), but were relatively well functioning in some other capacity domains (e.g. competence to judge and decide, Table 1 and Supplementary Table S4).

### Mini-ICF-APP ratings of claimants with depressive episodes

There were 204 participants with either mild, moderate, or severe depressive episodes (Table 2). The MICF_mean_ across these claimants was 1.46 (SD 0.59). The univariate ANOVA revealed that all Mini-ICF-APP ratings varied between the three diagnoses, with increased severity of the diagnosis being associated with higher Mini-ICF-APP ratings, as verified in post-hoc tests (Table 2 and Supplementary Table S5). The percentage of claimants with an RWC ≤ 30% was considerably larger in claimants with severe depressive episodes, as compared to claimants with mild and moderate depressive episodes (Table 2, bottom row).

### Relationship between Mini-ICF-APP ratings and RWC estimates

The Mini-ICF-APP ratings were compared between the claimants of the three subsamples, as defined by the estimated RWC in an alternative job. Across all participants with RWC estimates (*n* = 940), the RWC was on average 50.6% (SD 29.4%). The initial univariate ANOVA revealed significant differences in all Mini-ICF-APP domains, with (as expected) higher Mini-ICF-APP ratings being associated with lower RWC (Table 3). The rating profiles (reflecting the differential Mini-ICF-APP item difficulty) within each of the three RWC groups were very similar: All three subsamples exhibited the highest ratings for endurance and flexibility and the lowest ratings for self-care and mobility (Table 3), similar to previous reports [15]. Of note, relatively strong limitations in endurance were observed even in claimants with a high RWC: Almost 70% of the claimants with high RWC were rated at least as moderately limited in this domain (ratings ≥2, Table S6).

#### Regression analyses

**Fig. 1:**
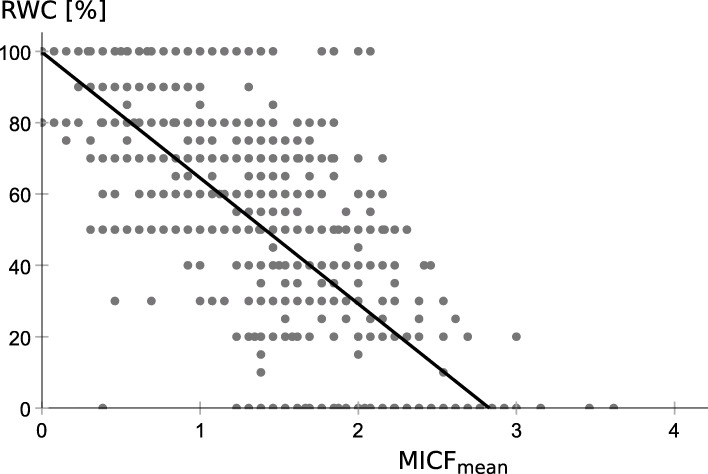
Simple linear regression model with MICF_mean_ as predictor. Scatterplot of the data, with the MICF_mean_ ratings on the x-axis and the RWC, as estimated by the medical expert, on the y axis. The black line represents the regression curve with MICF_mean_ as predictor for the RWC. This curve fit is compromised by heteroscedasticity, all data points with RWC = 100% are for example on the right side of the curve

In the initial simple linear regression analysis, we observed a strong association between the RWC and mean Mini-ICF-APP ratings (MICF_mean_, R^2^ = 0.518, Fig. 1, Table 4 top row). The stepwise multiple regression revealed a slightly higher R^2^ when four (or more) domain-specific ratings were entered as predictors, whereby the improvement of R^2^ was below 0.01 after entering a fifth predictor. Such model with five predictors encompassed the ratings for flexibility, endurance, intimate relationships, planning and structuring a task, and group integration (Table 4, middle row, R^2^ = 0.550). In a combined hierarchical multiple regression model, we first entered MICF_mean_ as predictor and subsequently entered the five domain-specific capacity ratings (as identified in the stepwise multiple regression) as additional predictors. In this model, the improvement of R^2^ was below 0.01 after entering endurance and intimate relationships as additional predictors (Table 4, bottom row, R^2^ = 0.548).

**Table 4:** Summary of the regression models

	Regression coefficient B	SE (B)	Standardized B	T Value	P value
**Simple linear regression:** R^2^ = 0.518					
Constant	99.779				
(I) MICF_mean_	−35.276	1.112	−0.720	59.913	< 0.001
**Stepwise multiple regression:** R^2^ = 0.550					
Constant	106.918	2.204		49.736	< 0.001
(I) MICF_flexibility_	−5.351	1.077	−.150	−4.967	< 0.001
(II) MICF_endurance_	−9.645	1.002	−.245	−9.630	< 0.001
(III) MICF_intimate relationships_	−6.579	.871	−.204	−7.555	< 0.001
(IV) MICF_planning and structuring of tasks_	−7.570	.927	−.235	−8.164	< 0.001
(V) MICF_group integration_	−5.619	.877	−.183	−6.406	< 0.001
**Hierarchical multiple regression:** R^2^ = 0.548					
Constant	108.212	2.066		52.379	< 0.001
(I) MICF_mean_	−26.365	1.579	−.537	−16.693	< 0.001
(II) MICF_endurance_	−7.060	1.070	−.178	−6.600	< 0.001
(III) MICF_intimate relationships_	−4.324	.896	−.133	−4.825	< 0.001

The table provides the summary of three regression models, calculated for predicting the RWC based on Mini-ICF-APP ratings. These were 1) the initial simple linear regression analysis (MICFmean as sole predictor, top section), 2) the stepwise multiple regression (with MICF ratings in the individual domains as predictors, middle section), and 3) the hierarchical stepwise regression (combining the first two approaches, bottom section)

**Fig. 2:**
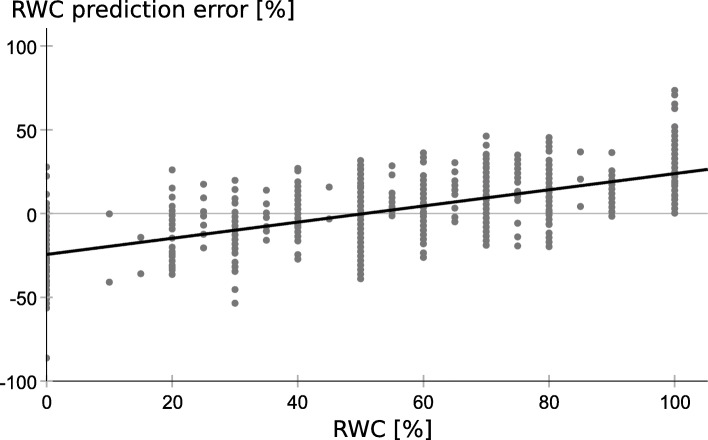
Systematic prediction error of the simple linear regression model with MICF_mean_ as predictor. Scatterplot of the prediction error for RWC in dependence of the RWC, reported by the medical expert to the insurer. The plot indicates that high RWCs were underestimated and low RWCs were overestimated, when predicted based on the MICF_mean_ score, as compared to the reported RWC.

**Fig. 3:**
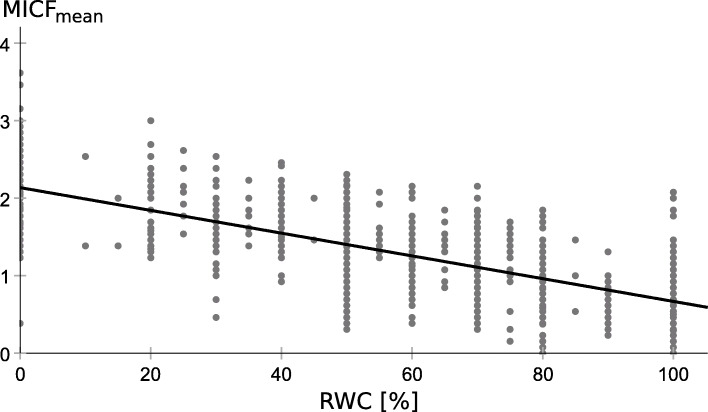
Simple linear regression model with RWC as predictor. Scatterplot of the data, with the RWC, as estimated by the medical expert, on the x axis and MICF_mean_ ratings on the y axis. The data are thus identical to those displayed in Fig. 1, with just the axes swapped. The black line represents the regression curve, with RWC values used for predicting MICF_mean_

Unfortunately, all models are flawed by heteroscedasticity, meaning the residual variance is not equally distributed and it shows a systematic linear trend with the dependent variable, as shown for the simple linear regression model with MICF_mean_ as predictor (Fig. 2). Thus, the regression models as described in Table 4 inform about the magnitude of the association between Mini-ICF-APP ratings and RWC estimate, but the estimation of the regression curves is flawed by the bounded data distribution. A more reliable regression curve can be obtained by reversing the dependent and independent variables, meaning to predict MICF_mean_ based on the RWC estimate (Fig. 3). (One could equally well argue that high Mini-ICF-APP ratings should be associated with low RWC estimates and that a low RWC should result in high Mini-ICF-APP ratings.) The data displayed in Fig. 3 is the very same data as displayed in Fig. 1, with just x and y axes swapped. The regression curve in Fig. 3 is less steep than one would expect from Fig. 1, but it now runs at each RWC level through the data points (MICF_mean predicted_ = − 0.015 * RWC + 2.136, R^2^ = 0.518).

In a subsequent explorative analysis, we compared the MICF_mean_ values between 10% levels of RWC in a univariate ANOVA, followed by post-hoc pairwise comparisons, in order to reveal how well these RWC levels could be differentiated based on the MICF_mean_. To this end, the values of RWC were rounded to multiples of 10%. The MICF_mean_ difference between 100 and 0% RWC was 1.49 (SE 0.06), meaning for each RWC decrease by 10% there was on average a MICF_mean_ increase by 0.15, which corresponds to approximately 2 points for MICF_total_. MICF_mean_ varied highly significantly between the eleven rounded levels of RWC (F _10, 929_ = 100.155, *P* < 0.001). However, the MICF_mean_ differences between two neighboring RWC levels varied between as little as −.02 (SD 0.08, 30 and 40% RWC) and as much as 0.29 (SD 0.06, 40 and 50%, Table 5). The MICF_mean_ differences between 20, 30, and 40% RWC, 60 and 70% RWC, as well as between 90 and 100% RWC did not reach statistical significance in the post-hoc testing.

**Table 5:** MICF_mean_ and MICF_total_ for each 10% level of RWC

	High RWC				Moderate RWC			Low RWC			
RWC_rounded_	100	90	80	70	60	50	40	30	20	10	0
MICF_mean_ (SD)	0.60 (0.50)	0.73 (0.30)	0.98 (0.42)	1.19 (0.44)	1.28 (0.35)	1.40 (0.40)	1.69 (0.33)	1.67 (0.47)	1.85 (0.48)	1.96 (0.82)	2.10 (0.44)
MICF_total_ (SD)	7.9 (6.6)	9.5 (4.0)	12.7 (5.4)	15.4 (5.7)	16.6 (4.6)	18.2 (5.2)	22.0 (4.3)	21.7 (6.1)	24.0 (6.2)	25.5 (10.6)	27.2 (5.8)
N	70	28	123	98	97	229	64	42	33	2	154

The categorical levels of high, moderate, and low RWC are marked in addition to the 10% levels. Due to the rounding of the RWC values for the purpose of this statistical comparison, the samples of high, moderate, and low RWC minimally vary between Tables 3 and 5

## Discussion

The study sought to analyze the difference in Mini-ICF-APP capacity limitation ratings between claimants with different psychiatric diagnoses and to determine how well the expert’s RWC estimate could be inferred from his corresponding Mini-ICF-APP ratings. The major findings are discussed below.

### Mini-ICF-APP ratings and psychiatric diagnoses

The comparison of the Mini-ICF-APP ratings between claimants with personality, mood, and neurotic disorders showed that the activity and participation limitations were most severe in claimants with personality disorders. Study participants with personality disorders particularly showed strong social limitations (contact with others, group integration, intimate relationships, adherence to regulations). This finding is in line with views that impairments in social, occupational, or other major areas of life represent an integral aspect of personality disorders [25, 26]. Yet, also in other domains (such as flexibility and endurance), the psychiatrists rated them as more limited, with flexibility generally considered as fundamental aspect of health, but also of social functioning [27]. Social interactions have been regarded as constituting element of work [28] and it is hard to imagine any kind of work which lacks this element. Given the degree and the kind of their capacity limitations, it was little surprising that experts assigned low RWCs to claimants with personality disorders more than twice as frequently than to claimants with mood or neurotic disorders (Table 1).

Nevertheless, the severity of capacity limitations in claimants with personality disorder was unexpected, also given the high prevalence of personality disorders in the general population [29]. Claimants with personality disorder and less severe capacity limitations appear to be underrepresented in the study sample. It is not fully clear why this could had been the case. It would be worrying if in order to qualify as ‘candidate’ for disability benefits the threshold of capacity limitations would be higher for individuals with personality disorders than for other disorders, and if less severe capacity limitations of such individuals would be downplayed and attributed to their difficult personality (rather than to their disorder). Future studies should pay special attention to capacity limitations in individuals with personality disorders to clarify this issue.

Among claimants with depressive episodes, the deficits increased across all domains with the severity of the diagnosis. This was expected as the severity of depressive episodes is largely defined by a) the symptom load (with more and more distressing symptoms present with increasing severity), and b) the ability to continue activities. The symptom load can be expected to affect activities and participation, as some symptoms of depressive episodes describe likewise limitations in function and activity/participation, as e.g. the reduction of energy. The finding shows that for claimants with depressive episodes the extent of limitations in activity and participation largely agreed with their psychiatric diagnosis. In contrast to the differences between claimants with personality disorders and claimants with mood and neurotic disorders, rating profiles of the three sub-samples with depressive episodes were quite similar. This finding is in line with previous studies, showing an association between MICF_total_ and the severity of the psychiatric symptoms [19, 20].

When placing the here investigated Mini-ICF-APP capacity limitations into context with previous research, it is important to consider the inclusion criteria of our study. The Mini-ICF-APP sum score of our participants with depressive episodes was on average 3 to 4 points higher than the scores for community-based patient samples with such diagnosis [16, 19]. Likewise, the sum score of the total sample was more than 7 points higher than the sum score of the sample reported in the Mini-ICF-APP manual, consisting of 213 psychosomatic rehabilitation patients [15]. The ratings in our sample corresponded to those observed for psychiatric inpatients after admission [20]. The on average high ratings in our study sample presumably reflect the fact that all study participants filed disability benefits. This step is usually proceeded only after return-to-work programs failed, implying persisting capacity deficits [30].

To sum up, aside from increased ratings with increased severity of the mental disorder, the current study revealed for the first time disorder-specific alterations of the Mini-ICF-APP capacity limitation ratings, with claimants with personality disorders showing pronounced limitations particularly in their social functioning.

### Mini-ICF-APP ratings and work-capacity ratings

In line with previous reports [15, 22, 23, 31], Mini-ICF-APP ratings showed significant differences between claimants with high, moderate, and low RWC (Table 4), which documents a consistency of expert estimates in RWC with observed limitations in activity and participation on a group level. Across all capacity domains, claimants with low RWC had the highest and those with high RWC the lowest Mini-ICF-APP ratings. The regression analyses showed, as expected, that the RWC linearly decreased with increasing Mini-ICF-APP ratings. This finding is well in line with another Swiss study that revealed a strong linear relationship between the Mini-ICF-APP sum score and work incapacity (as complement to work capacity [23]). However, in both studies, the estimation of the exact linear relationship is evidently hampered by heteroscedasticity (an unequal distribution of the residual variance).

In our study, inspection of the residual variance showed that high RWCs were underestimated and low RWCs were overestimated when using MICF_mean_ as predictor in the linear regression analysis (Fig. 2). For the curve fit, this leads to the impression of a mirrored z-form, with the regression curve as diagonal slash (Fig. 1). Such a z-form is similarly present in Fig. 2 of Habermeyer et al. [23]. Heteroscedasticity cannot be easily dissolved as it is related to the data distribution: As evident from Fig. 1**,** the RWC variance is considerably lower for MICF_mean_ ratings < 1 and > 2 than for MICF_mean_ ratings between 1 and 2. Related and likewise importantly, the assigned RWC values are bounded as the RWC can neither be worse than 0% nor better than 100%.

To tackle the problem of heteroscedasticity, we swapped the dependent and independent variable. The swapping resulted in a linear curve fit with a similar level of residual MICF_mean_ variance for each level of RWC (Fig. 3). To some extent, this regression curve can be used for estimating the plausibility of an assigned RWC based on MICF_mean_. However, Figs. 1 and 3 illustrate that empirically MICF_mean_ ratings between 1 and 2 were associated with any RWC (0 to 100%). This indicates that, even though the statistical correlation between MICF_mean_ and RWC was relatively high, it was not high enough to derive an individual’s RWC from his/her MICF_mean_ rating with sufficient confidence. About half of the RWC variance was unrelated to the capacity limitations documented in the Mini-ICF-APP although the very same medical expert provided the Mini-ICF-APP ratings and RWC estimate. One could argue that, when estimating the RWC, medical experts considered not just the amount of limitations, but also their kind, and they weighted limitations in some domains higher than in others. However, the multiple regression analysis did not reveal evidence that the consideration of domain-specific ratings resulted in a noticeably better RWC prediction than just considering MICF_mean_ as predictor.

The poor predictive power of the capacity-specific Mini-ICF-APP data might be to some extent due to the limited number of response options, which range from “0” (no disability) to “4” (total disability), with the latter response option being hardly ever applied in our sample (Supplement Table S3). The limited number of response options implies that the variance of ratings in each domain is limited, which constrains the explanatory power of these ratings as predictors for RWC as more finely graded outcome variable. Moreover, the usability of the Mini-ICF-APP ratings as predictors is restricted by how the Mini-ICF-APP ratings refer to the quantity of limitations: A rating of “0” refers to a range of 4% limitations (0 to 4%), whereas a rating of “3” refers to a range of 45% limitation (50 to 95%, [15, 16]). For rehabilitation and therapy, a coarse categorization of limitations might be considered as sufficient, because all limitations would anyway require a qualitative (rather than a quantitative) specification.

However, for the purpose of work disability evaluations, the Mini-ICF-APP should allow for a more finely graded rating. This raises the question whether it is possible to rate capacities like assertiveness based on clinical interviews and medical and other records with sufficiently high precision. Moreover, what is a sufficiently high precision? For RWC ratings, most stakeholders in Switzerland expect a maximum acceptable difference between two raters between 10 and 20% [32]. In a naturalistic study setting, Kunz et al. [7] showed that the RWC disagreements between two experts were > 20% in approximately one third of the evaluations, even though the medical experts received additional training beforehand. Thus, for Mini-ICF-APP ratings, a precision between 10 and 20% would be an optimistic expectation. Ideally, the grading of the Mini-ICF-APP ratings should correspond to the commonly used 10% RWC levels, when these ratings are supposed to serve the purpose to document capacity limitations contributing to a diminished RWC. However, to what degree psychiatric experts are able to reliably discriminate 10% differences in their capacity ratings of individual Mini-ICF-APP items when using clinical judgment needs to be tested empirically.

An essential aspect for determining the role of the Mini-ICF-APP in work disability evaluations is how the instrument is actually used in this context. The manual suggests that just 10 min are required to provide the ratings and to analyze the results. This does not correspond to the perceptions in insurance medicine [33]. These authors stress that for work disability evaluations first the relevant information needs to be gathered from different sources and subsequently checked for consistency. They recommend that the ratings should be accompanied by a “narrative explanation”, informing about based on which information the expert provided his rating. Such narrative explanations would increase the transparency and (to some extent) the plausibility of the evaluation, as the information relevant for the evaluation is documented, like it is suggested for injury-related disability evaluations [34]. For an example, see Kunze [35]. We propose that ideally these narrative explanations refer to the job demands in the last job as well as in an alternative job, and are based on functional interviews [34, 36].

To sum up, RWC estimates cannot be derived from Mini-ICF-APP ratings with sufficient precision, even when considering capacity-specific ratings. About half of the RWC variance was unrelated to activity and participation limitations documented in the Mini-ICF-APP ratings. This large variability only enables a plausibility check of RWC estimates and the identification of gross outliers (values in far distance to the regression curve, as depicted in Fig. 3).

### Study limitations

The reported data were obtained in a large sample of claimants for disability benefits in a real-life setting. As one major study limitation, the number of medical experts as participants was quite limited. Given that three of them conducted more than 80% of the ratings, the individual rating behavior could have had some impact on the results, even though the profiles of the Mini-ICF-APP ratings presented here are generally well in accordance with previous reports [15, 22, 23, 31]. For example, there could have been some variance between experts how they conceptualized the individual domains of the Mini-ICF-APP and limitations herein. Moreover, even though all participating experts worked in the same assessment center and likely shared some standards, there might still have been systematic differences between them. These differences may have included the preference for diagnosing certain psychiatric disorders over others, a focus on specific limitations during exploration, or how finely graded their RWC estimation was. Future studies should include a larger number of medical experts in order to minimize the impact of individual rater characteristics on the results, even though this would not necessarily improve the RWC prediction based on the Mini-ICF-APP ratings. However, a detailed analysis of an expert’s rating profile might provide some valuable feedback for the expert himself in order to identify in which aspects his rating behavior varies from the rating behavior of others (e.g. by being too conservative or too liberal in the ratings).

The basic concept of the current study is that activity and participation limitations can be translated into an RWC estimate. However, the specification and quantification of such limitations in the work context are not trivial, even though anchor points for the rating are clearly defined in the Mini-ICF-APP manual. The current study just considers the endpoints: namely, on the one hand the activity and participation limitations as rated in the Mini-ICF-APP, and on the other hand the RWC, as estimated by the expert. The process of the formation of these values was not considered in this study. Given this, the study does not provide insights about from which sources the RWC variance stems that is unrelated to the Mini-ICF-APP ratings. It might stem from fuzzy ratings, maybe related to variable, inconsistent limitations of the claimant, to insufficient knowledge of medical experts about work and work requirements, or related to the lack of reliable information at hand, e.g. when a claimant was out of work for an already considerable period. However, it might also stem from imprecise RWC estimates, as previous studies showed considerable variance between experts for the very same case [5–7].

## Conclusions

The Mini-ICF-APP allows the documentation of activity and participation limitations of claimants with psychiatric disorders. If accompanied by narrative explanations, these ratings can help stakeholders (including claimants, case managers, treating physicians, lawyers, judges etc.) to understand in condensed form based on what observations the medical expert estimated the RWC. The Mini-ICF-APP ratings thus provide a bridge between the psychiatric diagnosis and RWC, with various mental activity and participation domains systematically considered. The consistency between the Mini-ICF-APP capacity limitation ratings and RWC is likely to increase if the claimants’ work-related limitations are assessed in the light of the demands of particular jobs and occupations. Given this, medical experts should always report MICF_mean_, capacity-specific ratings, *and* provide narrative explanations. Our study showed that it is possible, in principle, to assess the plausibility of RWC estimates based on the mean Mini-ICF-APP rating as well as to check the plausibility of a claimant’s Mini-ICF-APP profile with regard to his or her psychiatric diagnosis. However, the considerable variance in Mini-ICF-APP ratings at each RWC level as well as within psychiatric diagnoses implies that only gross inconsistencies would stand out as evidently implausible data.

## Supplementary Information

**Additional file 1 Table S1** Study sample. **Table S2** Mean Mini-ICF-APP ratings across all participants. **Table S3** Distribution of the Mini-ICF-APP ratings across the total sample. **Table S4** Mini-ICF-APP ratings for the different psychiatric diagnoses. **Table S5** Mini-ICF-APP ratings for claimants with depressive episodes. **Table S6** Mini-ICF-APP ratings for the three remaining work capacity (RWC) levels.

## Data Availability

Data are available from the senior author (JJ) upon reasonable request.

## Abbreviations

RWC: remaining work capacity
ICD: International Classification of Diseases
ANOVA: analysis of variance
ICF: International Classification of Functioning, Disability and Health
APP: activity and participation limitations in psychiatric diseases [‘*Aktivitäts- und Partizipationsbeeinträchtigungen bei psychischen Erkrankungen*’]
MICF: Mini-ICF-APP
MICF_mean_: mean rating in the Mini-ICF-APP
MICF_total_: summed rating score in the Mini-ICF-APP
