# Supplementary material for: The assessment of capacity limitations in psychiatric work disability evaluations by the social functioning scale Mini-ICF-APP
Source: BMC Psychiatry. 2021 Sep 30;21:480. doi: 10.1186/s12888-021-03467-w (PMC8485557; doi:10.1186/s12888-021-03467-w)
Supplement: Supplementary file 1 — Additional file 1 Table S1 Study sample. Table S2 Mean Mini-ICF-APP ratings across all participants. Table S3 Distribution of the Mini-ICF-APP ratings across the total sample. Table S4 Mini-ICF-APP ratings for the different psychiatric diagnoses. Table S5 Mini-ICF-APP ratings for claimants with depressive episodes. Table S6 Mini-ICF-APP ratings for the three remaining work capacity (RWC) levels. [file 12888_2021_3467_MOESM1_ESM.docx]

**The assessment of capacity limitations in psychiatric work disability evaluations by the social functioning scale Mini-ICF-APP**

**Timm Rosburg**^1^**, Regina Kunz**^1^**, Bruno Trezzini**^2,3^**, Urban Schwegler**^2,3^**, Jörg Jeger**^4^

**Affiliations**

^1^ University of Basel Hospital, Department of Clinical Research, EbIM, Research & Education, Basel, Switzerland

^2^ Swiss Paraplegic Research, Nottwil, Switzerland

^3^ University of Lucerne, Department of Health Sciences and Medicine, Lucerne, Switzerland

^4^ MEDAS Zentralschweiz, Lucerne, Switzerland

***Corresponding author:**

E-mail: [timm.rosburg@usb.ch](mailto:timm.rosburg@usb.ch)**Table S1** *Study sample*

|  | All | | Females | | Males | |
| --- | --- | --- | --- | --- | --- | --- |
|  | N | Age  (SD) | N | Age  (SD) | N | Age  (SD) |
| Total sample | 946 | 48.9  (8.5) | 532 | 48.0  (8.3) | 414 | 50.2  (8.5) |
| Mood disorders (F30-F39)  *Including* | 359 | 50.1  (7.7) | 192 | 49.3  (7.1) | 167 | 51.1  (8.2) |
| Mild depressive episode (F32.0) | 58 | 51.3  (7.4) | 25 | 51.7  (6.1) | 33 | 50.9  (8.3) |
| - Moderate depressive episode (F32.1) | 112 | 49.8  (7.3) | 60 | 49.5  (6.8) | 52 | 50.1  (7.9) |
| - Severe depressive episode (F32.2) | 34 | 47.7  (7.3) | 21 | 47.6  (6.4) | 13 | 47.8  (8.0) |
| Neurotic, stress-related and somatoform disorders (F40-F48) | 347 | 48.9  (8.5) | 218 | 47.6  (8.7) | 129 | 51.1  (7.6) |
| Disorders of adult personality and behaviour (F60-F69) | 142 | 47.4  (8.8) | 80 | 46.4  (8.7) | 62 | 48.7  (8.9) |
| Psychiatric diagnoses other than listed above | 96 | 46.6  (9.8) | 40 | 46.4  (9.3) | 56 | 46.7  (10.2) |

Distribution of the primary psychiatric diagnoses in the study sample, stratified by sex, and mean age (SD) [in years]. The diagnosis was missing in two cases.

**Table S2 Mean Mini-ICF-APP ratings across all participants**

|  | Mean | SD | N |
| --- | --- | --- | --- |
| (1) adherence to regulations | 1.39 | .94 | 946 |
| (2) planning and structuring of tasks | 1.45 | .90 | 946 |
| (3) flexibility | 1.96 | .82 | 946 |
| (4) applying expertise | 1.30 | .92 | 945 |
| (5) competence to judge and decide | 1.43 | .99 | 928 |
| (6) endurance | 2.17 | .74 | 946 |
| (7) assertiveness | 1.67 | .90 | 946 |
| (8) contact with others | 1.35 | .92 | 946 |
| (9) group integration | 1.46 | .96 | 946 |
| (10) intimate relationships | 1.28 | .91 | 945 |
| (11) non-work activities | 1.56 | .85 | 946 |
| (12) self care | .30 | .61 | 946 |
| (13) mobility | .77 | .92 | 946 |
| MICF_mean_ | 1.39 | .60 | 946 |
| MICF_total_ | 18.09 | 7.76 | 946 |

**Table S3 Distribution of the Mini-ICF-APP ratings across the total sample**

|  | “0”  No  disability | “1”  Mild  disability | “2”  Moderate disability | “3”  Severe disability | “4”  Total disability |
| --- | --- | --- | --- | --- | --- |
| (1) adherence to regulations | 21.0 % | 27.7 % | 40.2 % | 9.9 % | 0.3 % |
| (2) planning and structuring of tasks | 18.3 % | 28.9 % | 42.3 % | 10.4 % | 0.1 % |
| (3) flexibility | 6.7 % | 15.1 % | 54.4 % | 23.0 % | 0.7 % |
| (4) applying expertise | 22.3 % | 33.8 % | 35.4% | 8.0 % | 0.4 % |
| (5) competence to judge and decide | 23.3 % | 24.0 % | 39.9 % | 12.5 % | 0.3 % |
| (6) endurance | 3.0 % | 10.7 % | 53.3 % | 32.0 % | 1.0 % |
| (7) assertiveness | 12.2 % | 25.6 % | 44.7 % | 17.4 % | 0.1 % |
| (8) contact with others | 19.9 % | 36.8 % | 32.1 % | 10.9 % | 0.3 % |
| (9) group integration | 18.0 % | 32.5 % | 35.1 % | 13.8 % | 0.6 % |
| (10) intimate relationships | 21.6 % | 37.8% | 31.7 % | 8.6 % | 0.3 % |
| (11) non-work activities | 10.7 % | 35.4 % | 41.4 % | 11.0 % | 0.6 % |
| (12) self care | 76.5 % | 17.8 % | 4.8 % | 0.8 % | 0.1 % |
| (13) mobility | 50.1% | 28.2 % | 16.8 % | 4.1 % | 0.7 % |

**Table S4 Mini-ICF-APP ratings for the different psychiatric diagnoses**

|  | “0”  No disability | “1”  Mild disability | “2”  Moderate disability | “3”  Severe disability | “4”  Total disability |
| --- | --- | --- | --- | --- | --- |
| **(1) Adherence to regulations** | | | | | |
| F30-F39 | 20.1% | 30.9% | 39.8% | 8.6% | 0.6% |
| F40-F48 | 29.7% | 30.8% | 34.6% | 4.9% | 0.0% |
| F60-F69 | 13.4% | 14.8% | 46.5% | 25.4% | 0.0% |
| **(2) Planning and structuring of tasks** | | | | | |
| F30-F39 | 13.9% | 29.2% | 44.8% | 12.0% | 0.0% |
| F40-F48 | 25.9% | 32.3% | 36.6% | 4.9% | 0.3% |
| F60-F69 | 17.6% | 26.8% | 41.5% | 14.1% | 0.0% |
| **(3) Flexibility** | | | | | |
| F30-F39 | 3.3% | 13.6% | 60.2% | 22.3% | 0.6% |
| F40-F48 | 13.0% | 17.3% | 55.3% | 13.8% | 0.6% |
| F60-F69 | 2.1% | 12.7% | 40.8% | 43.0% | 1.4% |
| **(4) Applying expertise** | | | | | |
| F30-F39 | 16.8% | 41.3% | 34.9% | 6.4% | 0.6% |
| F40-F48 | 29.7% | 33.4% | 30.5% | 6.1% | 0.3% |
| F60-F69 | 23.9% | 22.5% | 38.7% | 14.8% | 0.0% |
| **(5) Competence to judge and decide** | | | | | |
| F30-F39 | 17.3% | 22.2% | 45.0% | 15.0% | 0.6% |
| F40-F48 | 29.3% | 28.4% | 35.9% | 6.4% | 0.0% |
| F60-F69 | 30.0% | 22.1% | 30.7% | 17.1% | 0.0% |
| **(6) Endurance** | | | | | |
| F30-F39 | 1.7% | 8.6% | 61.6% | 26.5% | 1.7% |
| F40-F48 | 3.5% | 11.0% | 57.5% | 27.5% | 0.6% |
| F60-F69 | 3.5% | 12.7% | 31.0% | 52.1% | 0.7% |
| **(7) Assertiveness** | | | | | |
| F30-F39 | 7.2% | 20.1% | 54.0% | 18.4% | 0.3% |
| F40-F48 | 19.9% | 34.3% | 34.6% | 11.2% | 0.0% |
| F60-F69 | 9.9% | 19.0% | 47.9% | 23.2% | 0.0% |
| **(8) Contact with others** | | | | | |
| F30-F39 | 17.0% | 35.7% | 40.1% | 7.0% | 0.3% |
| F40-F48 | 27.7% | 47.3% | 19.9% | 4.9% | 0.3% |
| F60-F69 | 5.6% | 22.5% | 40.8% | 30.3% | 0.7% |
| **(9) Group integration** | | | | | |
| F30-F39 | 17.3% | 35.9% | 38.7% | 7.5% | 0.6% |
| F40-F48 | 24.8% | 38.9% | 30.8% | 4.6% | 0.9% |
| F60-F69 | 2.8% | 14.1% | 38.0% | 45.1% | 0.0% |
| **(10) Intimate relationships** | | | | | |
| F30-F39 | 18.1% | 42.1% | 33.4% | 6.4% | 0.0% |
| F40-F48 | 30.0% | 40.6% | 22.2% | 6.6% | 0.6% |
| F60-F69 | 9.9% | 24.8% | 44.0% | 20.6% | 0.7% |
| **(11Non-work activities** | | | | | |
| F30-F39 | 9.5% | 36.8% | 43.5% | 9.2% | 1.1% |
| F40-F48 | 11.5% | 40.6% | 39.8% | 7.5% | 0.6% |
| F60-F69 | 10.6% | 18.3% | 48.6% | 22.5% | 0.0% |
| **(12) Self care** | | | | | |
| F30-F39 | 78.0% | 16.4% | 4.7% | 0.8% | 0.0% |
| F40-F48 | 81.3% | 14.7% | 3.5% | 0.6% | 0.0% |
| F60-F69 | 66.9% | 25.4% | 6.3% | 1.4% | 0.0% |
| **(13) Mobility** | | | | | |
| F30-F39 | 50.4% | 24.8% | 19.2% | 5.0% | 0.6% |
| F40-F48 | 48.7% | 31.7% | 16.7% | 2.0% | 0.9% |
| F60-F69 | 54.2% | 27.5% | 12.0% | 5.6% | 0.7% |

Distribution of the Mini-ICF-APP ratings for the three sub-samples with different ICD-F diagnoses. The percentage values refer to the sample described in the row.

**Table S5 Mini-ICF-APP ratings for claimants with depressive episodes**

|  | “0”  No disability | “1”  Mild disability | “2”  Moderate disability | “3”  Severe disability | “4”  Total disability |
| --- | --- | --- | --- | --- | --- |
| **(1) Adherence to regulations** | | | | | |
| F32.0 | 46.6% | 41.4% | 12.1% | 0.0% | 0.0% |
| F32.1 | 12.5% | 33.9% | 50.0% | 3.6% | 0.0% |
| F32.2 | 14.7% | 11.8% | 41.2% | 29.4% | 2.9% |
| **(2) Planning and structuring of tasks** | | | | | |
| F32.0 | 37.9% | 32.8% | 29.3% | 0.0% | 0.0% |
| F32.1 | 5.4% | 33.0% | 58.9% | 2.7% | 0.0% |
| F32.2 | 5.9% | 11.8% | 41.2% | 41.2% | 0.0% |
| **(3) Flexibility** | | | | | |
| F32.0 | 6.9% | 27.6% | 62.1% | 3.4% | 0.0% |
| F32.1 | 0.0% | 6.3% | 76.8% | 17.0% | 0.0% |
| F32.2 | 2.9% | 2.9% | 26.5% | 64.7% | 2.9% |
| **(4) Applying expertise** | | | | | |
| F32.0 | 29.3% | 56.9% | 13.8% | 0.0% | 0.0% |
| F32.1 | 8.0% | 46.4% | 41.1% | 4.5% | 0.0% |
| F32.2 | 5.9% | 23.5% | 38.2% | 29.4% | 2.9% |
| **(5) Competence to judge and decide** | | | | | |
| F32.0 | 35.1% | 29.8% | 31.6% | 3.5% | 0.0% |
| F32.1 | 10.1% | 21.1% | 60.6% | 8.3% | 0.0% |
| F32.2 | 6.9% | 3.4% | 41.4% | 41.4% | 6.9% |
| **(6) Endurance** | | | | | |
| F32.0 | 3.4% | 15.5% | 77.6% | 3.4% | 0.0% |
| F32.1 | 0.9% | 3.6% | 73.2% | 21.4% | 0.9% |
| F32.2 | 0.0% | 0.0% | 38.2% | 52.9% | 8.8% |
| **(7) Assertiveness** | | | | | |
| F32.0 | 13.8% | 25.9% | 56.9% | 3.4% | 0.0% |
| F32.1 | 0.9% | 20.5% | 67.9% | 10.7% | 0.0% |
| F32.2 | 2.9% | 5.9% | 52.9% | 38.2% | 0.0% |
| **(8) Contact with others** | | | | | |
| F32.0 | 36.2% | 43.1% | 20.7% | 0.0% | 0.0% |
| F32.1 | 6.3% | 44.6% | 46.4% | 2.7% | 0.0% |
| F32.2 | 2.9% | 14.7% | 58.8% | 20.6% | 2.9% |
| **(9) Group integration** | | | | | |
| F32.0 | 36.2% | 43.1% | 20.7% | 0.0% | 0.0% |
| F32.1 | 7.1% | 42.0% | 46.4% | 3.6% | 0.9% |
| F32.2 | 5.9% | 14.7% | 55.9% | 20.6% | 2.9% |
| **(10) Intimate relationships** | | | | | |
| F32.0 | 29.3% | 44.8% | 25.9% | 0.0% | 0.0% |
| F32.1 | 7.1% | 44.6% | 42.0% | 6.3% | 0.0% |
| F32.2 | 2.9% | 32.4% | 44.1% | 20.6% | 0.0% |
| **(11) Non-work activities** | | | | | |
| F32.0 | 31.0% | 46.6% | 22.4% | 0.0% | 0.0% |
| F32.1 | 0.9% | 42.0% | 49.1% | 8.0% | 0.0% |
| F32.2 | 2.9% | 5.9% | 64.7% | 20.6% | 5.9% |
| **(12) Self care** | | | | | |
| F32.0 | 94.8% | 5.2% | 0.0% | 0.0% | 0.0% |
| F32.1 | 78.6% | 17.9% | 3.6% | 0.0% | 0.0% |
| F32.2 | 50.0% | 26.5% | 23.5% | 0.0% | 0.0% |
| **(13) Mobility** | | | | | |
| F32.0 | 86.2% | 12.1% | 1.7% | 0.0% | 0.0% |
| F32.1 | 50.9% | 20.5% | 22.3% | 6.3% | 0.0% |
| F32.2 | 26.5% | 23.5% | 38.2% | 8.8% | 2.9% |

Distribution of the Mini-ICF-APP ratings for the three sub-samples with depressive episodes (F32.0 to F32.2). The percentage values refer to the sample described in the row.

**Table S6 Mini-ICF-APP ratings for the three remaining work capacity (RWC) levels**

|  | “0”  No disability | “1”  Mild disability | “2”  Moderate disability | “3”  Severe disability | “4”  Total disability |
| --- | --- | --- | --- | --- | --- |
| **(1) Adherence to regulations** | | | | | |
| RWC ≤ 30 % | 6.9% | 12.6% | 45.9% | 33.3% | 1.3% |
| RWC >30 %, <70 % | 15.2% | 29.6% | 52.0% | 3.2% | 0.0% |
| RWC ≥70 % | 41.4% | 37.5% | 20.5% | 0.7% | 0.0% |
| **(2) Planning and structuring of tasks** | | | | | |
| RWC ≤ 30 % | 4.3% | 14.7% | 45.0% | 35.5% | 0.4% |
| RWC >30 %, <70 % | 11.4% | 31.8% | 54.0% | 2.7% | 0.0% |
| RWC ≥70 % | 37.5% | 36.5% | 25.1% | 1.0% | 0.0% |
| **(3) Flexibility** | | | | | |
| RWC ≤ 30 % | 0.9% | 5.2% | 33.3% | 57.6% | 3.0% |
| RWC >30 %, <70 % | 3.2% | 10.7% | 70.4% | 15.7% | 0.0% |
| RWC ≥70 % | 16.0% | 28.3% | 50.5% | 5.2% | 0.0% |
| **(4) Applying expertise** | | | | | |
| RWC ≤ 30 % | 7.8% | 15.7% | 50.9% | 23.9% | 1.7% |
| RWC >30 %, <70 % | 16.9% | 37.8% | 41.8% | 3.5% | 0.0% |
| RWC ≥70 % | 39.4% | 43.0% | 15.6% | 2.0% | 0.0% |
| **(5) Competence to judge and decide** | | | | | |
| RWC ≤ 30 % | 15.5% | 12.4% | 40.3% | 30.5% | 1.3% |
| RWC >30 %, <70 % | 18.6% | 25.9% | 45.8% | 9.6% | 0.0% |
| RWC ≥70 % | 34.4% | 31.1% | 31.4% | 3.0% | 0.0% |
| **(6) Endurance** | | | | | |
| RWC ≤ 30 % | 0.4% | 4.8% | 26.8% | 64.1% | 3.9% |
| RWC >30 %, <70 % | 1.0% | 5.2% | 62.7% | 31.1% | 0.0% |
| RWC ≥70 % | 7.2% | 22.9% | 62.1% | 7.8% | 0.0% |
| **(7) Assertiveness** | | | | | |
| RWC ≤ 30 % | 3.9% | 9.5% | 46.8% | 39.4% | 0.4% |
| RWC >30 %, <70 % | 9.2% | 23.6% | 53.5% | 13.7% | 0.0% |
| RWC ≥70 % | 22.1% | 40.7% | 32.9% | 4.2% | 0.0% |
| **(8) Contact with others** | | | | | |
| RWC ≤ 30 % | 4.3% | 19.0% | 44.2% | 31.2% | 1.3% |
| RWC >30 %, <70 % | 14.4% | 43.8% | 36.6% | 5.2% | 0.0% |
| RWC ≥70 % | 39.1% | 41.4% | 18.2% | 1.3% | 0.0% |
| **(9) Group integration** | | | | | |
| RWC ≤ 30 % | 4.3% | 10.8% | 47.2% | 35.5% | 2.2% |
| RWC >30 %, <70 % | 12.7% | 35.8% | 42.0% | 9.2% | 0.2% |
| RWC ≥70 % | 35.2% | 44.6% | 18.9% | 1.3% | 0.0% |
| **(10) Intimate relationships** | | | | | |
| RWC ≤ 30 % | 3.0% | 26.0% | 44.6% | 26.0% | 0.4% |
| RWC >30 %, <70 % | 17.7% | 41.6% | 36.4% | 4.2% | 0.0% |
| RWC ≥70 % | 40.4% | 43.0% | 15.6% | 0.3% | 0.7% |
| **(11) Non-work activities** | | | | | |
| RWC ≤ 30 % | 2.2% | 17.3% | 51.1% | 26.8% | 2.6% |
| RWC >30 %, <70 % | 4.7% | 36.6% | 50.0% | 8.7% | 0.0% |
| RWC ≥70 % | 24.4% | 48.2% | 25.4% | 2.0% | 0.0% |
| **(12) Self care** | | | | | |
| RWC ≤ 30 % | 51.5% | 31.6% | 13.4% | 3.0% | 0.4% |
| RWC >30 %, <70 % | 81.3% | 15.9% | 2.5% | 0.2% | 0.0% |
| RWC ≥70 % | 89.6% | 9.4% | 1.0% | 0.0% | 0.0% |
| **(13) Mobility** | | | | | |
| RWC ≤ 30 % | 35.5% | 30.7% | 22.9% | 7.8% | 3.0% |
| RWC >30 %, <70 % | 46.0% | 29.4% | 20.9% | 3.7% | 0.0% |
| RWC ≥70 % | 66.1% | 25.1% | 7.2% | 1.6% | 0.0% |

Distribution of the Mini-ICF-APP ratings for the three sub-samples defined by their different RWC levels. The percentage values refer to the sample described in the row.
